# Ticagrelor Versus Clopidogrel in Patients With Acute Coronary Syndrome and on Dialysis: A Meta-Analysis

**DOI:** 10.7759/cureus.40211

**Published:** 2023-06-10

**Authors:** Venkata Sai Harshabhargav Chenna, Hemalatha Anam, Majid Hassan, Abdul Moeez, Raja Reddy, Sandipkumar S Chaudhari, Koushik Sapkota, Muhammad Usama

**Affiliations:** 1 Medicine, University of Perpetual Help System Dalta, Las Pinas, PHL; 2 Medicine, Apollo institute of Medical Sciences and Research, Hyderabad, IND; 3 Medicine, Universidad Autonoma de Guadalajara, Sacramento, USA; 4 Medicine, Services Hospital Lahore, Lahore, PAK; 5 Medicine, MNR Medical College and Hospital, Hyderabad, IND; 6 General Practice, Lions General Hospital, Mehsana, IND; 7 General Practice, Gujarat Medical Education and Research Society (GMERS) Medical College and Hospital, Vadnagar, IND; 8 Medicine, All India Institute of Medical Sciences (AIIMS) Bathinda, Bathinda, IND; 9 Neurology, Sheikh Zayed Medical College/Hospital Rahim Yar Khan, Rahim Yar Khan, PAK

**Keywords:** efficacy, dialysis, acute coronary syndrome, ticagrelor, clopidogrel

## Abstract

This study aims to compare the safety and efficacy of clopidogrel and ticagrelor in patients with acute coronary syndrome (ACS) and undergoing dialysis. This study was conducted per the guidelines of the Preferred Reporting of Systematic Reviews and Meta-Analyses (PRISMA). A comprehensive search was performed using electronic databases, including PubMed, EMBASE, and Web of Science, to identify relevant studies comparing clopidogrel and ticagrelor in patients undergoing dialysis. To ensure the inclusion of all relevant articles, a combination of the following keywords, along with medical subject heading (MeSH) terms, was used: "clopidogrel," "ticagrelor," "acute coronary syndrome," and "dialysis." The primary endpoint of this meta-analysis was the incidence of major adverse cardiovascular events (MACE), which consisted of cardiovascular death, myocardial infarction, stroke, and revascularization. The secondary endpoint was all-cause mortality. The occurrence of any bleeding events (including major and nonmajor bleeding events) and major bleeding events was chosen as the safety endpoints. A total of four studies were included in the pooled analysis. The pooled sample size was 5,417 patients, including 892 in the ticagrelor group and 4525 in the clopidogrel group. The findings indicate that ticagrelor, compared to clopidogrel, is associated with a significantly higher risk of MACEs, all-cause death, and major bleeding events. The findings suggest that clopidogrel may be a better choice for individuals with ACS undergoing dialysis due to its lower risk of MACE, all-cause death, and major bleeding events compared to ticagrelor.

## Introduction and background

Cardiovascular conditions account for approximately 33% of global mortality, leading to around 7.5 million deaths attributed to ischemic heart disease (IHD). Acute coronary syndromes (ACSs) and sudden death are the primary causes of IHD-related fatalities, accounting for approximately 1.8 million deaths annually [1]. Chronic kidney disease (CKD) and end-stage renal disease (ESRD) are major risk factors for cardiovascular illness, and the risk increases as renal function deteriorates [2]. Not only do CKD and ESRD accelerate the development of coronary artery disease (CAD), but they also impact its symptoms and clinical manifestations [3]. CKD is present in 30% to 40% of patients presenting with myocardial infarction (MI) [4-5], and it is associated with an increased risk of recurrent MI, all-cause mortality, cardiovascular mortality, and bleeding complications [6-7].

The 2016 guidelines from the American College of Cardiology/American Heart Association recommend that patients with acute MI (AMI) should take both aspirin and a specific type of oral P2Y12 receptor antagonist for one year [8]. In the past, clopidogrel was the most commonly used P2Y12 receptor antagonist for AMI patients [9]. However, new P2Y12 receptor antagonists have been developed specifically for ACS. The Platelet Inhibition and Patient Outcomes (PLATO) trial demonstrated that ticagrelor, one of these new antagonists, is associated with lower rates of MI and cardiovascular death compared to clopidogrel, without an increase in major bleeding incidents [10]. According to current guidelines, ticagrelor is recommended over clopidogrel for AMI patients. Nevertheless, patients undergoing dialysis are not included in these recommendations due to the lack of clinical evidence [11].

Patients with ESRD exhibit altered platelet function compared to the general population, resulting in a higher tendency for bleeding and a hypercoagulation state [12-13]. Several studies have demonstrated that patients undergoing dialysis experience worse outcomes following percutaneous coronary intervention (PCI), and even after successful PCI [14-15], one-year mortality rates remain elevated in dialysis patients [16]. Unlike clopidogrel, ticagrelor exhibits a more rapid onset and offset of platelet inhibition effect, even in patients receiving hemodialysis, and it is not influenced by the cytochrome P450 (CYP) 2C19 allele [17]. However, it is still unknown whether the antiplatelet effect of ticagrelor can reduce thrombotic events or increase bleeding events in ESRD patients. Currently, there is a lack of studies comparing the clinical outcomes of ticagrelor and clopidogrel specifically in patients receiving dialysis for ACS. Therefore, it is important to conduct a pooled analysis of available literature to compare the outcomes in patients undergoing dialysis. This meta-analysis aims to compare the safety and efficacy of clopidogrel and ticagrelor in patients with ACS and undergoing dialysis.

## Review

Methodology

This study was conducted in accordance with the guidelines of the Preferred Reporting of Systematic Reviews and Meta-Analyses (PRISMA). A comprehensive search was performed using electronic databases, including PubMed, EMBASE, and Web of Science, to identify relevant studies comparing clopidogrel and ticagrelor in patients undergoing dialysis from inception to May 15, 2023. To ensure the inclusion of all relevant articles, a combination of the following keywords, along with medical subject headings (MeSH) terms, was used: "clopidogrel," "ticagrelor," "acute coronary syndrome," and "dialysis." Additionally, we manually searched the reference lists of all included studies to avoid the omission of any relevant articles in the present meta-analysis.

Inclusion and Exclusion Criteria

Eligible studies were required to fulfill the inclusion criteria of adult patients affected by ACS and undergoing dialysis, and to have performed comparisons between clopidogrel and ticagrelor. We included studies that reported the outcomes assessed in this meta-analysis. Reviews, case reports, case series, and editorials were excluded from the meta-analysis. Studies published in languages other than English were also excluded. Two authors independently reviewed the eligible records. After removing duplicates, screening was performed based on titles and abstracts. The full text of all eligible records was obtained, and a detailed assessment using predefined inclusion and exclusion criteria was conducted. Any disagreements between the two authors were resolved through consensus.

Data Extraction and Quality Assessment

Two authors independently reviewed all included records using standardized data-abstraction forms created in Microsoft Excel. The data extracted from the included studies included the author's name, publication year, sample size, follow-up duration, baseline demographics, and clinical outcomes during the follow-up period. The quality of the included studies was assessed using the Newcastle-Ottawa Scale. Any disagreements between the two authors in the process of data extraction and quality assessment were resolved through consensus.

Study Endpoints

The primary endpoint of this meta-analysis was the incidence of major adverse cardiovascular events (MACEs), which consisted of cardiovascular death, MI, stroke, and revascularization. The secondary endpoint was all-cause mortality. The occurrence of any bleeding events (including major and nonmajor bleeding events) and major bleeding events was chosen as the safety endpoints.

Statistical Analysis

The meta-analysis was performed using RevMan Version 5.4.1 (The Cochrane Collaboration, London, UK). The endpoints were recorded as dichotomous variables, and comparisons were made using hazard ratios (HR) and 95% confidence intervals (CIs). Statistical significance was considered when *P* < 0.05. Heterogeneity among study results was reported using the *I*^2 ^statistics and Cochran's Q statistic. If the *P*-value of the Cochran's Q test was less than 0.10 and/or the *I*^2 ^statistic was ≥50%, significant heterogeneity was considered, and a random effects model was used. Otherwise, a fixed-effect model was used. We performed sensitivity analysis using a jackknife approach.

Results

**Figure 1:**
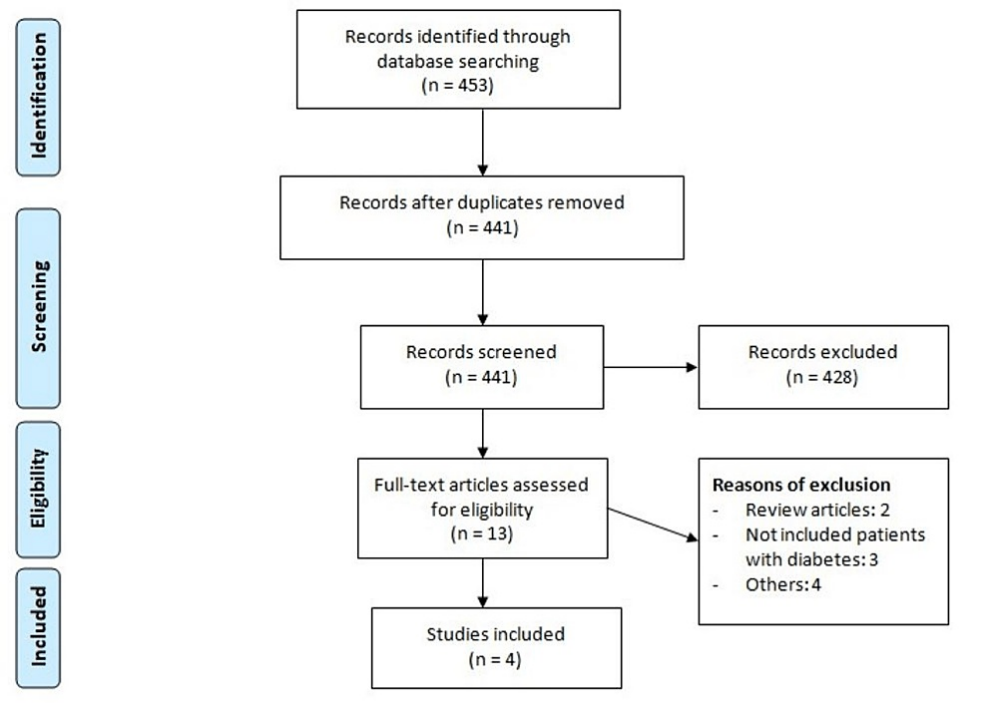
PRISMA flowchart of study selection. PRISMA, Preferred Reporting of Systematic Reviews and Meta-Analyses

Figure 1 shows process of study selection. Total 453 citations were obtained through electronic databases. After removing duplicates, initial screening of 441 studies was done using title and abstracts. Thirteen studies were eligible for full-text assessment. Finally, four studies met the inclusion criteria and included in the final meta-analysis. Table 1 shows the characteristics of included studies. Pooled sample size was 5,417 patients, including 892 in ticagrelor group and 4,525 in the clopidogrel group. Table 2 presents the quality assessment.

**Table 1:** Characteristics of included studies. NR, not reported

Author name	Year	Study design	Region	Groups	Sample size	Follow-up (Months)	MI (%)	Mean age (Years)	Males (%)
Chen et al. [18]	2022	Cohort	China	Ticagrelor	18	12	NR	NR	NR
				Clopidogrel	32				
Lee et al. [19]	2019	Cohort	Switzerland	Ticagrelor	74	12	13.50	66.5	62.1
				Clopidogrel	116				
Li et al [20]	2020	Cohort	Taiwan	Ticagrelor	270	12	80.70%	66.8	57.4
				Clopidogrel	1,915				
Tung et al. [21]	2021	Cohort	Taiwan	Ticagrelor	530	9	100	NR	57.3
				Clopidogrel	2,462				

**Table 2:** Quality assessment.

Author Names	Selection	Comparison	Outcome	Overall
Chen et al. [18]	4	2	2	Good
Lee et al. [19]	3	2	2	Good
Li et al. [20]	4	2	2	Good
Tung et al. [21]	3	2	2	Good

Major Adverse Cardiovascular Events

**Figure 2:**
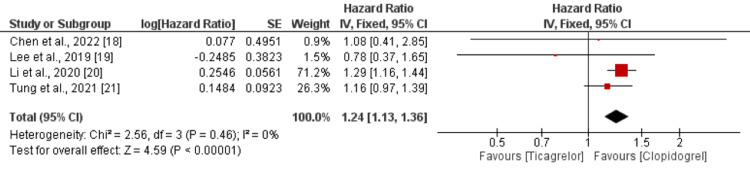
Major adverse cardiovascular events. Sources: [18-21]. CI, confidence interval; SE, standard error

Four studies compared risk of MACE between the ticagrelor group and the clopidogrel group. Pooled analysis showed that risk of MACE was higher in the ticagrelor group than the clopidogrel group (HR = 1.24; 95% CI = 1.13-1.36; *P*-value < 0.001), as shown in Figure 2. No heterogeneity was reported among the study results (*I*^2 ^= 0%).

As shown in Table 3, the overall incidence of cardiovascular death was significantly higher in the ticagrelor group compared to the clopidogrel group (HR = 1.60; 95% CI = 1.38-1.86; *I*^2 ^= 45%). Similarly, the risk of MI was not significantly different between the ticagrelor group and the clopidogrel group (HR = 0.94; 95% CI = 0.77-1.14; *I*^2 ^= 0%). The risk of stroke was lower in patients receiving ticagrelor, but the difference is statistically insignificant (HR = 0.62; 95% CI = 0.24-1.58; *I*^2 ^= 54%).

**Table 3:** Separate analysis of MACEs. ^^^Significant at *P*-value <0.05. HR, hazard ratio; CI, confidence interval; MACE, major adverse cardiovascular event

Outcomes	HR (95% CI)	*P*-value	*I*^2^
Cardiovascular death	1.60 (1.38-1.86)	0.001^^^	45%
Myocardial infarction	0.94 (0.77-1.14)	0.51	0%
Stroke	0.62 (0.24-1.58)	0.32	54%

All-Cause Death

**Figure 3:**
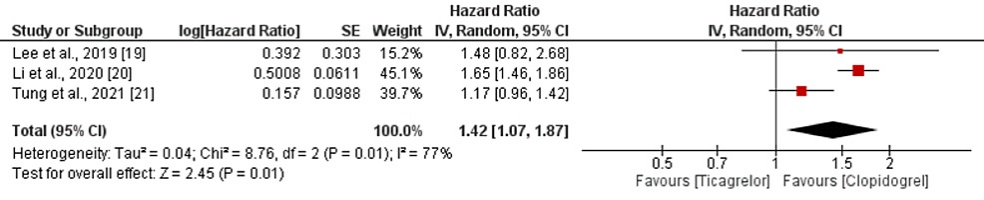
All-cause death. Sources: [19-21]. CI, confidence interval; SE, standard error

Three studies were included in the pooled analysis of comparison of all-cause mortality between clopidogrel group and ticagrelor group. As shown in Figure 3, a lower risk of all-cause death was observed in the clopidogrel group compared to the ticagrelor group (HR = 1.42; 95% CI = 1.07-1.87). Significant heterogeneity was reported among the study results (*I*^2 ^= 77%).

Safety Analysis

**Figure 4:**
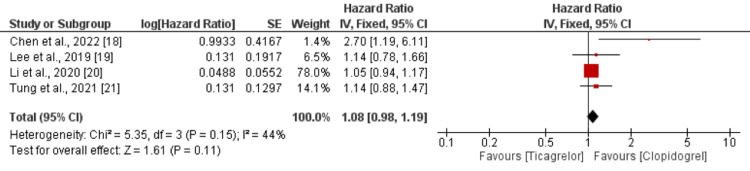
Bleeding events. Sources: [18-21]. CI, confidence interval; SE, standard error

**Figure 5:**
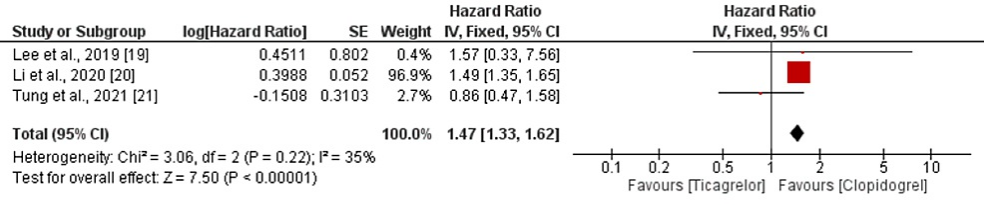
Major bleeding events. Sources: [19-21]. CI, confidence interval; SE, standard error

As shown in Figure 4, the risk of all bleeding events was higher in patients receiving ticagrelor compared to patients received clopidogrel. However, the difference was statistically insignificant (HR = 1.08; 95% CI = 0.98-1.19). No significant heterogeneity was reported among the study results (*I*^2 ^= 44%). In terms of major bleeding events, the risk is significantly higher in the ticagrelor group compared to the clopidogrel group (HR = 1.47; 95% CI = 1.33-1.62), as shown in Figure 5. No significant heterogeneity was reported among the study results (*I*^2 ^= 35%).

Sensitivity Analysis

We performed sensitivity analysis using a jackknife approach whereby each study is removed individually to test the robustness of the pooled HR with 95% CI. As shown in Table 4, when excluding study conducted by Li et al. [20], no significant differences were reported in terms of MACE. However, in relation to all-cause mortality, results were consistent across subgroups. 

**Table 4:** Sensitivity analysis. MACE, major adverse cardiovascular event; HR, hazard ratio; CI, confidence interval

Study	HR (95% CI)	*I*^2^
MACE		
All studies included	1.24 (1.13-1.36)	0%
Chen et al. [18]	1.24 (1.13-1.37)	19%
Lee et al. [19]	1.25 (1.14-1.37)	0%
Li et al. [20]	1.13 (0.95-1.35)	0%
Tung et al. [21]	1.27 (1.14-1.42)	0%
All-cause death		
All studies included	1.42 (1.07-1.87)	77%
Lee et al. [19]	1.40 (1.01-1.96)	89%
Li et al. [20]	1.20 (1.01-1.44)	0%
Tung et al. [21]	1.64 (1.46-1.85)	0%
Any bleeding events		
All studies included	1.08 (0.98-1.19)	44%
Chen et al. [18]	1.07 (0.97-1.18)	0%
Lee et al. [19]	1.08 (0.98-1.19)	62%
Li et al. [20]	1.20 (0.98-1.47)	50%
Tung et al. [21]	1.22 (0.87-1.71)	61%
Major bleeding events		
All studies included	1.47 (1.33-1.62)	35%
Lee et al. [19]	1.23 (0.74-2.06)	67%
Li et al. [20]	0.93 (0.53-1.64)	0%
Tung et al. [21]	1.49 (1.35-1.65)	0%

Discussion

To the best of our knowledge, this meta-analysis represents the first comparison of the effects of ticagrelor and clopidogrel on patients with ACS undergoing dialysis. The findings revealed that ticagrelor, when compared to clopidogrel, was associated with a significantly higher risk of MACE, all-cause death, and major bleeding events. Consequently, clopidogrel may be considered a more favorable option for individuals with ACS and undergoing dialysis. However, it is important to interpret these findings with caution due to the retrospective nature of the included cohort studies and the limitations associated with pooled analyses.

Among the four studies that compared the incidence of MACE between ticagrelor and clopidogrel patients, three studies reported a higher risk of MACE in the ticagrelor group. Notably, the study conducted by Lee et al. [19] reported a lower incidence of MACE in the ticagrelor group, although the difference was statistically insignificant, potentially due to a lower number of events compared to the other included studies. Moreover, the pooled analysis of MACE heavily relied on the study by Li et al. [20]. However, sensitivity analysis performed after excluding this particular study showed no significant difference between the two groups in terms of MACE. Previous studies have consistently shown that patients on dialysis face an increased risk of MACE compared to the general population [20]. This finding is not surprising, as cardiovascular disease accounts for 22% of fatalities in individuals with normal kidney function, while in those experiencing renal failure, 71% of deaths can be attributed to cardiovascular disease [22]. The dialysis process itself can contribute to the development of atherosclerosis by promoting platelet creation and accumulation, complement factors, and polymorphonuclear cells. Oxidation products present during dialysis may impair endothelial function, further exacerbating the risk of atherosclerosis. Additionally, impaired kidney function can lead to elevated blood phosphorus levels and hinder the effectiveness of dialysis. Elevated phosphorus levels prompt an increase in serum calcium levels, ultimately resulting in the calcification of blood vessels [23]. Past studies have consistently reported a mortality risk 10 to 30 times higher in patients undergoing dialysis compared to the general population [24-25].

The majority of studies included in this meta-analysis did not observe a consistent advantage of ticagrelor over clopidogrel in reducing the risk of cardiovascular events among patients with ESRD undergoing dialysis. In a Swedish study mentioned earlier, the data indicated that ticagrelor had a lower risk of MACE compared to clopidogrel in all estimated glomerular filtration rate (eGFR) groups, except for patients with an eGFR below 30 mL/minute, where the CI was wide and crossed the unity line [10]. These findings suggest that ticagrelor's ability to prevent subsequent cardiovascular events may diminish as kidney function deteriorates progressively. However, conclusive statements cannot be made based solely on this meta-analysis, and future randomized controlled trials (RCTs) are needed to compare the effectiveness of ticagrelor and clopidogrel in reducing the risk of cardiovascular events among dialysis patients.

The risk of major bleeding events in patients with ESRD is predicted to be 20 times higher than in patients with normal renal function [26]. Despite differences in follow-up duration among studies, our analysis found that the risk of major bleeding events was significantly higher in patients receiving ticagrelor. Several factors contribute to ticagrelor's higher risk of bleeding events. First, ticagrelor exhibits a faster onset of action and more potent antiplatelet effects compared to other antiplatelet medications like clopidogrel. This increased potency can lead to a greater inhibition of platelet function and subsequently raise the risk of bleeding [27]. Therefore, patients undergoing dialysis should be aware of the associated bleeding symptoms when using ticagrelor. A study conducted by Mavrakanas et al. utilizing the United States Renal Data System analyzed the outcomes of P2Y12 inhibitors in dialysis patients and reported that ticagrelor was comparable to clopidogrel in terms of ischemic outcomes but associated with a higher incidence of bleeding events [28].

The current meta-analysis has several limitations. First, it did not analyze individual patient data. Second, only four studies were included, and none of them were RCTs. Additionally, one study carried most of the weight in the pooled analysis of outcomes. Therefore, caution must be exercised when interpreting the findings. Finally, there were no definite maintained durations or dosages of ticagrelor for these patients, which could have influenced the final results. In the future, larger RCTs need to be conducted to compare the optimal therapy for ACS in patients undergoing dialysis.

## Conclusions

In this meta-analysis, we compared the effects of ticagrelor and clopidogrel on patients with ACS undergoing dialysis. Four studies met the inclusion criteria and were included in the final analysis. The findings indicate that ticagrelor, compared to clopidogrel, is associated with a significantly higher risk of MACE, all-cause death, and major bleeding events. The findings suggest that clopidogrel may be a better choice for individuals with ACS undergoing dialysis due to its lower risk of MACE, all-cause death, and major bleeding events compared to ticagrelor. However, these conclusions are based on a pooled analysis of retrospective cohort studies, and caution should be exercised when interpreting the results. Patients with ESRD undergoing dialysis are known to have an increased risk of major bleeding events. Ticagrelor's higher risk of bleeding events can be attributed to its faster onset of action and more potent antiplatelet effects. Patients on dialysis should be aware of bleeding symptoms while using ticagrelor.
